# Adaptation of colorectal cancer screening tailored navigation content for American Indian communities and early results using the intervention

**DOI:** 10.1186/s43058-022-00253-x

**Published:** 2022-01-28

**Authors:** Usha Menon, Peter Lance, Laura A. Szalacha, Dianna Candito, Emily P. Bobyock, Monica Yellowhair, Jennifer Hatcher

**Affiliations:** 1 College of Nursing, University of South Florida, 12901 Bruce B. Downs Blvd, MDC Box 22, Tampa, FL 33612 USA; 2 University of Arizona Cancer Center, 1515 N. Campbell Ave, Tucson, AZ 85724 USA; 3 Morsani College of Medicine, University of South Florida, 12901 Bruce B. Downs Blvd, Tampa, FL 33612 USA

**Keywords:** American Indian (AI), Colorectal cancer screening, Implementation science, Tailored patient navigation

## Abstract

**Background:**

American Indians (AI) experience major colorectal cancer (CRC) screening disparities with commensurate inequity in CRC mortality and other outcomes. The purpose of this report is to describe the methods and early results of adapting a previously successful intervention for the AI community.

**Methods:**

The educational content and delivery strategy of the parent intervention were adapted for AIs guided by an adaptation framework and cultural consultations with the community and clinicians. As part of the environmental scanning, we identified the need to substantively revise our data entry, collection, and tracking system and develop a REDCap database for this purpose. In this study, we staggered the implementation of the intervention in each facility to inform the process from one clinic to the next, and assess both the clinical outcomes of the tailored intervention and the implementation processes across two clinic settings, Facilities A and B.

**Results:**

The REDCap database is an indispensable asset, and without it we would not have been able to obtain reliable aggregate screening data while improvements to facility electronic health records are in progress. Approximately 8% (*n* = 678) of screening-eligible patients have been exposed to the navigator intervention. Of those exposed to the navigator intervention, 37% completed screening.

**Conclusions:**

With the small numbers of patients exposed so far to the intervention, it would be premature to draw any broad conclusions yet about intervention effects. However, early screening completion rates are substantial advances on existing rates, and we have demonstrated that a tailored navigator intervention for facilitating CRC screening was readily adapted with provider and community input for application to AIs. A REDCap database for tracking of CRC screening by navigators using tablets or laptops on- or offline is easy to use and allows for generation of aggregate, anonymized screening data.

Trial registration.

There was no health intervention meeting the criteria of a clinical trial. The University of Arizona Institutional Review Board granted exemption from obtaining informed consent from patients undergoing CRC screening after administration of the tailored navigation intervention as usual care.

**Supplementary Information:**

The online version contains supplementary material available at 10.1186/s43058-022-00253-x.

Contributions to the literature

- Successful interventions can rarely be implemented as-is in a practice setting but with adaption may be successful.
- Adaptation should include tailoring to the clinic setting and population to be served.
- Iterative formative testing is imperative because even clinics within one healthcare system may have different implementation needs.

## Background

Although still the second leading cause of death from cancer in the United States (US), overall colorectal cancer (CRC) incidence and mortality at the population level have declined steadily in recent decades in men and women over the age of 50 years [1, 2]; screening is largely credited for these declines [1, 3–6]. However, over the same period, such declines in CRC incidence and mortality have conspicuously evaded American Indians and Alaska Natives (AI/AN) [5, 7–9]. Further, AI/AN have some of the lowest CRC screening rates in the US [10–12]. Roughly two-thirds of US adults are up-to-date with previous US Preventive Services Task Force (USPSTF) guidelines which recommended that CRC screening begin at age 50 [13–15]; in contrast, the Indian Health Service (IHS) reported that for the years 2018 and 2019, respectively, only 31.9% and 31.5% of AI/AN patients nationally had undergone appropriate CRC screening [16]. Because of increasing CRC incidence rates in individuals below the age of 50, in May 2021, USPSTF recommended that the age at which CRC screenings begin should be lowered to 45 [17].

The American Indian (AI) CRC Screening Consortium was formed by the National Cancer Institute (NCI)-designated Cancer Centers at the Universities of Arizona, New Mexico, and Oklahoma to use an implementation science approach to begin to rectify these glaring AI CRC screening health disparities. The Consortium’s University of Arizona Cancer Center (UACC) component is the focus of this report. Because the focus of the consortium is on AI only, we will use that term going forward to describe our study population, while background literature review will reference AI/AN where appropriate.

A role for patient navigators has been forcefully advocated in the elimination of various health disparities, [18] and a recent scoping review [19] identified multiple reports of significant increases in CRC screening rates that were achieved through the use of patient navigators [20–25]. Concerns about the impracticability and costs of providing universal individual patient-level navigator services to promote CRC screening adherence prompted development of system-level interventions for this purpose linked to the electronic health record (EHR). Automated mailing of fecal immunochemical test (FIT) kits features prominently in these efforts [26, 27]. The combination of mailed FIT kits and phone follow-up has been shown to sustain increased adherence to CRC screening compared to usual care without these measures over a period of years [28, 29].

The voluminous CRC screening literature is essentially devoid of reports of navigator-based and other interventions to increase screening rates specifically among AI/AN populations. Unique circumstances of many AI/AN communities and the consequent lack of applicability of strategies that successfully increase CRC screening in other segments of the US population compound the urgency of correcting this deficiency.

The US Postal Service (USPS) does not deliver to homes in many remote parts of AI/AN tribal lands, including the service area of a Public Law 93–638 facility participating in the current study (see below); mailed FIT programs are inoperable for patients who lack USPS home delivery. Many parts of tribal lands lack cell phone coverage, and the areas lacking USPS delivery and cell phone coverage often coincide; phone and text messaging approaches to increase CRC screening adherence are redundant in the absence of cell phone (or landline) service. Language and other cultural barriers are major impediments to CRC screening uptake among AI/AN [30].

We report here the methods and results of adapting a navigation-based intervention and the early outcomes of the intervention on AI CRC screening disparities in an environment, as described, that hampers strategies found to be effective in the larger US population. A tailored navigation intervention that was developed and tested previously by our group [31–33] was adapted for AIs. The TIDieR (Template for Intervention Description and Replication) guide and checklist were used to describe the adapted intervention in sufficient detail (see Additional file 1) [34].

## Methods

### Study design and population

The study applies a navigator-based intervention, specifically tailored to AI (see below), to enhance screening among AI at average risk for CRC using an interrupted time-series design [35]. At the time the study was designed and for the period of implementation reported here, USPSTF defined those at average risk for CRC as all men and women between the ages of 50 and 75 years without additional factors putting them at increased risk for the disease [13]. Patient navigators who are fluent in the language of their tribe were engaged at two AI-serving clinics in Arizona, one a Public Law 93–638 facility on tribal land (Facility A) and the other an urban Federally Qualified Health Center (FQHC, Facility B). Facilities A and B, respectively, provide care for approximately 5,800 and 1,200 AI men and women between the ages of 50 and 75 years. All these screening-eligible patients were considered potential targets for the study intervention, without exclusions. Navigators contacted eligible patients individually by phone or in person at the clinic.

### CRC screening approach and methods

Our purpose is to enhance rates of CRC screening by whichever USPSTF-recommended methods providers at participating facilities prefer, strictly as part of usual clinical care [13, 36]. At the time of study initiation and since, a fecal immunochemical test (FIT) is the primary screening test chosen by most providers for most patients, with screening colonoscopy the test of choice for a minority of patients. No health intervention meeting criteria for a clinical trial is involved. Therefore, exemption was granted by the University of Arizona Institutional Review Board (IRB) from obtaining informed consent from patients undergoing CRC screening at participating facilities while the study is active.

### Intervention Adaptation Framework

The adaptation of the intervention was guided by frameworks established by Card et al. [37] albeit with modification. Because we were leveraging an existing intervention, [31–33] we did not seek out other materials. Our original intervention was adapted in the five recommended areas: language (words, literacy, cultural references in the message library [TIMS^©^] and the training materials); updating research-based information (CRC and screening impact on AIs), images and exemplars for cultural relevance; updating staff training (all navigator training materials); and evaluation materials (REDCap database entry, navigator logs, EHR verifications). Below we describe the adaptations in more detail. The components of the original intervention and the adaptations are in Table 1.

**Table 1:** Original and Adapted Components of the Intervention

Intervention Component	Original	Adaptation	Adaptation details
**Setting**	Primary care clinics and community	Primary care clinics and community	
**Patient level tailored education**	Tailored Message Library	Tailored Message Library	Revised for culturally appropriate language, graphics, statistics
	Navigator Log	Navigator Log	
**Patient level—eligibility criteria**	Average and high-risk patients at average or high-risk for CRC for CRC screening	Average and high-risk patients at average or high-risk for CRC for CRC screening	Navigator training included talking with patients and barriers when referred for diagnostic testing
		Those referred for diagnostic testing follow-up	
**Patient level Intervention delivery**	In person or phone	In person or phone	Navigator training revised to address issues specific to the AI community
	Delivered by trained Navigator	Delivered by trained Navigator	
**Clinic level intervention**	None	Included	Feedback to clinic on CRC screening
			Use of REDCap for patient tracking
**Community level setting for education**	Urban and rural, Arizona	Primarily rural/reservation land, Arizona	
**Community level education content**	Awareness of CRC risk and screening	Awareness of CRC risk and screening	Revised to address issues specific to the AI community
**Implementation strategies**	Environmental scan	Environmental scan	Use of a newly developed REDCap database for logging navigation encounters, and tracking screening and diagnostic completion
	Navigators hired by study	Navigators hired by study	
	No EHR entry by navigators	EHR entry by navigators	

### The Tailored Intervention Messaging System^©^ (TIMS^©^)

TIMS^©^ is a computer-based, theory-driven educational program for increasing CRC screening rates [33] based on the Health Belief Model [38]. We developed and tested this tailored navigator intervention (TIMS^©^) in a randomized controlled trial design in a mixed African American and older non-Hispanic white population [33]. In a subsequent validating randomized controlled trial using TIMS^©^, conducted in an urban, underserved, largely Hispanic population, completion of CRC screening was increased almost fourfold in individuals receiving the tailored navigation intervention compared to the control [31].

### Adaptation of TIMS^©^ for an AI population

An AI Community Advisory Board (CAB) advises UACC on the cancer-related needs of tribal communities and prioritizes the relevance of proposed research projects to these communities. Endorsement of the project and suggestions for adaptation of TIMS^©^ for AIs were sought from the CAB. Approvals were obtained from the University of Arizona IRB and regulatory bodies for the two AI-serving study clinics to perform environmental scans at each facility. Informed consent was obtained from all participants in the scans, which took two forms: structured interviews—eight to ten at each facility—were conducted of individual providers, including physicians, administrators, nurses, nurse practitioners, medical assistants, and pharmacists; and focus groups of community members were conducted at each facility. Discussions were recorded and data were transcribed verbatim.

Environmental scan data were arranged in two categories: system implementation and patient factors. System implementation factors [35] were used to inform the assessment of implementation measures and to adapt the process of implementing the intervention in the clinic. Patient factors identified from the environmental scans were used as a lens through which to examine and modify the messaging library.

### Development of AI-adapted TIMS^©^ library

The CAB endorsed the project and suggested adaptations for the use of TIMS^©^ in AI communities. These suggestions and results from environmental scans at Facilities A and B were incorporated in the adapted library by two members of the study team experienced with using TIMS^©^ in communities and clinics [31, 32, 39]. Messages in the new library were divided into three categories (A – C):

A. **Messages in the existing library also identified in the current thematic analysis.** Messages in this category were retained with changes in wording suggested by the CAB and other AI cultural consultants. For example:
  ***Control health. Get screened to take control of health: It’s great that you want to take control of your health. Now take the next step and ask your doctor today about a stool blood test. Make a commitment to take control of your health***.
  ***Family health. Does not have a relative with colon cancer: You are at risk even if you don’t have a close relative with colon cancer. Many American Indian men and women who get colon cancer are like you and have a history of it in their family***.
B. **Messages in the existing library that were not identified in the current thematic analysis. **Messages in this category were reviewed by the CAB and other AI cultural consultants and revised to be suitable and idiomatic for AIs. For example:
  ***Religion – Original message. High- or low-risk because of religious faith: Men/women of all religious faiths get colon cancer. The important thing is to find the cancer early so that good medical treatment, as well as the power of prayer, will help you live a long life***.
  ***Revised message. High- or low-risk because of religious faith: Men/women of all religious faiths get colon cancer. Native peoples respect cultural beliefs and traditions of their tribes but may still get colon cancer. The important thing is to find the cancer early so that good medical treatment, as well as the power of faith, will assist in healing and recovery***.
  Although these issues did not arise in the environmental scans, they are retained with modifications in the message library because the qualitative interviews and focus groups could not be considered exhaustive.
C. **Messages not in the existing library that arose in the current thematic analysis.** Several issues of particular importance or unique to AIs were identified. For example, the use of “age-appropriate” staff or navigators was important; “elders” did not want to discuss important health issues with a “young person.” Poor communication and follow-up were other important issues; community members described calling to find out test results, not being able to reach the right providers, and not being able to find out their results. New messages for these community concerns were developed in conjunction with the CAB and other AI cultural consultants:
  ***Age-appropriate staff. Sometimes, it isn’t possible to have someone of your age available to talk with you about your health. I know this can feel uncomfortable, especially if the person is much younger than you. Everyone working in the clinic is trained to provide you with the best care. I’m also always available as your navigator to assist you***.
  ***Test results unavailable. Nearly all paper charts and records have been entered into paperless electronic health record systems. This allows providers to track your health records much more efficiently and accurately. We have also put in a system of checks to make sure that we follow up on your colon cancer screening results and arrange any further tests that may be necessary***.

### CRC screening rates at participating facilities

The Government Performance and Results Act (GPRA) requires healthcare facilities under the Federal umbrella, such as FQHCs and PL 93–638 facilities, to report performance data for CRC screening and other specified healthcare measures [40]. The GPRA CRC screening measure gives the percentage of AI patients aged 50–75 years who are up to date with CRC screening. The most recent GPRA rates for Facilities A and B were taken as the baseline rates at those facilities before introduction of the TIMS^©^ CRC screening navigator intervention.

We had initially crafted a study approach that depended heavily on extraction of aggregate anonymized EHR data to track CRC screening rates over time. Based on our preliminary environmental scan, the initially proposed data collection strategy and tools were rapidly revised to meet study needs and align with clinic capability and process (evaluation adaptation) [37]. Prior to implementation, we also created a REDCap database [41, 42] to serve as a backup for data collection during the initial stage of the project while we were familiarizing ourselves with facility EHRs. Navigators access this database on- or off-line on their tablet, laptop, and desktop computers. Steps in the screening process that are tracked by navigators in REDCap at the individual patient level include: administration of a FIT kit; return of the kit to the laboratory for development; FIT result, i.e., positive or negative; referral for colonoscopy of patients with a positive FIT; and completion and results of the diagnostic colonoscopy. Study investigators and staff have access only to deidentified, anonymized and aggregate data, including: the numbers of FIT kits distributed and returned; the percentages of positive and negative FITs; and the percentage of patients with a positive FIT who complete diagnostic colonoscopy.

### Navigator training

Navigators are bachelor’s prepared and have at least 2 years’ experience in clinic or community settings in AI communities. Training was conducted in person and, because of the Covid pandemic, necessarily more by videoconference than originally planned. Topics covered included: CRC biology, epidemiology – in the general US population and AIs, risk factors, diagnosis and treatment, AI CRC disparities, and screening – methods, results, and outcomes; behavioral and psychosocial aspects – stages of change and motivational interviewing; and patient navigation, which was similar to the original navigator training [31]. The only changes made were to the study funding information, clinic flow targeted to each separate clinic, use of the REDCap data and the navigator log (which was now contained in REDCap as opposed to software used in the other study). In the original study, all navigator training was in-person and role play was by phone. With COVID-19 travel restrictions, all navigator training was conducted over Zoom with additional breaks to account for web meeting fatigue. All role play was conducted by telephone.

Hands-on training was given on data entry into REDCap with mock patient material and screening results. One-on-one training was conducted by team leaders on how to integrate AI-adapted TIMS^©^ messages into the navigation process. This included the use of reflective listening and how to draw out barriers to screening, aided by a roadmap that provided cues for the navigation conversation. Finally, we developed standardized participant profiles, and navigators used role play with trainer feedback to solidify their expertise in delivering the navigation intervention.

While the interpersonal communication style used during the intervention was left up to each navigator, intervention standardization and fidelity was supported by: 1) Conducting group training which ensured that consistent information was given to all navigators regarding the protocol and process; 2) Using standardized participants during training role-play to deliver consistent situation-based training; 3) Evaluating training role play through a standardized scoring rubric and requiring all navigators to score 80 or above before proceeding into the field; 4) Having all navigators use a standardized manual of tailored messages on CRC-related knowledge, beliefs and barriers; and 5) Conducting regular meetings during the course of the formative stages of the study so that navigators and the project coordinator could review protocol and discuss unanticipated situations.

## Results

### Implementation of the navigator intervention tailored for AIs

The dosage of the navigation intervention was determined by each patient's needs. Navigators contacted patients up to six times. The median number of contacts was two. The duration of each interaction varied by the purpose of the contact (introduction, follow-up, reminder, etc.) but typically ranged from one to five minutes.

Facility A recruited a full-time study patient navigator who initiated the intervention and started data collection in November 2019. The Covid pandemic struck the Facility A community in February 2020 and all ambulatory clinics were completely closed because of the pandemic from mid-March through the end of August 2020. There was almost no CRC screening while the clinics were closed. Clinic patient visits continued to be much curtailed beyond August 2020, and clinic activity did not return to full capacity until the first months of 2021. Unavoidably severe impairment of navigator ability to reach eligible patients and have them undergo CRC screening continued and only abated as the Covid pandemic receded.

Facility B, which initiated the CRC screening project in January 2020, chose initially to divide CRC screening navigation between two existing staff members, who continued their previous tasks that were unrelated to CRC screening while assuming their new CRC screening-related responsibilities. Facility B clinics were closed because of Covid from March 2020 for the remainder of the year, and only opened again slowly in 2021. The original navigators, with their multiple responsibilities, reported difficulty prioritizing these diverse duties and were concerned that the quality of work on all their tasks was suffering. To remedy the situation, clinic administration recruited a new navigator in March 2021 from outside the organization. This full-time individual is devoted solely to CRC screening navigation.

### CRC screening rates after introduction of navigators

**Fig. 1:**
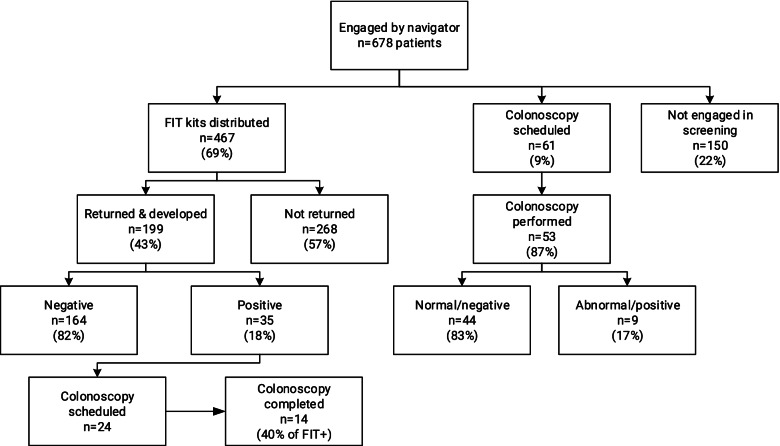
Navigator CRC screening activity

It was quickly apparent that facility EHR capabilities could not adequately track CRC screening at the individual patient and system levels. The REDCap database was therefore substituted as the primary tool for tracking CRC screening as an adaptation to the methods of data collection. Aggregate REDCap data for navigator-mediated CRC screening in eligible patients at Facilities A and B from November 2019 through August 2021 are shown in Fig. 1, including the Covid-interrupted period. The navigators interacted with a total of 678 patients during this time (575 at Facility A and 103 at Facility B), the great majority of these interactions occurring face-to-face in the course of non-emergency, scheduled clinic visits. Of the 678 patients, 150 (22%) did not engage in screening. Of 467 patients provided a FIT kit, 43% applied stool and returned the kits for development. A total of 252 patients (37% of those engaged by a navigator) completed screening by FIT (*n* = 199) or colonoscopy (*n* = 53). Of the 199 patients who returned a FIT kit to the laboratory for development, 18% tested positive and 82% negative. The GPRA rates for CRC screening at Facilities A and B, respectively, in September 2021 were 27% and 15%.

### Post-implementation adaptations

In addition to switching to REDCap as the primary method for data collection while EHR improvements are introduced, the environmental scan exposed an important barrier to screening and follow-up at Facility B. For primary screening, patients were being referred for colonoscopy to another facility 80 miles away and often did not complete the procedure for logistical reasons. Two adaptations have been introduced to work around this barrier. First, FIT is now the preferred primary screening modality. Second, arrangements have been made for a local community gastroenterology practice to accept patients referred from Facility B for diagnostic colonoscopy following a positive FIT.

## Discussion

As noted, USPSTF recommended (B Recommendation) in May 2021 that the threshold age to start CRC screening should be lowered to 45 years [36] from the previous threshold of 50 years. The earlier 50-year-old threshold was in operation over the period for which screening results are reported here, but both facilities from which results are reported are now targeting patients at average risk for CRC for screening from the age of 45 years.

We have now adapted the navigation intervention entirely (educational, tailored messages, the navigator training and documentation, data collection tools and data entry processes) to the specific needs of AIs, first through consultation with AI community leaders and cultural consultants and then through incorporation of findings from environmental scans at participating AI-serving facilities. The process of adapting the navigation intervention for AIs yielded substantive modifications and additions. An important takeaway is that an intervention of proven efficacy in one community or setting may not be immediately suitable for other communities and settings. But, equally important, adaptation of a well-tested strategy to a new setting is quite easily accomplished without the need to develop a new intervention from scratch. Besides tailoring of TIMS^©^ to AIs, several other useful adaptations have surfaced during this preliminary phase.

In light of the unanticipated EHR inadequacies, the REDCap database has proved an indispensable asset; without it we would not have been able to obtain reliable aggregate screening data while improvements to facility EHRs are in progress. The Covid pandemic is not implicated in this essential adaptation to the methods of project informatics. Approximately 8% (*n* = 678) of screening-eligible patients have been exposed to the navigator intervention at Facilities A and B. With the small numbers of patients exposed so far to the intervention, it would be premature to draw any broad conclusions yet about intervention effects. Nonetheless, given the baseline GPRA rates of only 9% and 13%, respectively, at Facilities A and B, it is encouraging that 37% of patients with whom a navigator has interacted have completed screening by FIT or colonoscopy. The increase in the current GPRA rate at Facility A to 27% is also encouraging, but the extent to which the intervention can be credited is uncertain.

Study facilities have strictly limited colonoscopy capability. Our suggestion to move to a strategy of FIT first followed by diagnostic colonoscopy for patients with a positive FIT and to restrict use of colonoscopy as the primary screening tool was well received. This adaptation has been adopted at a system level and is now the usual approach at both the facilities featured in this report. We have learned that at least during the start-up phase of introducing CRC screening navigation, navigators with additional duties to implementing the study intervention are at risk of neglecting this task. We have adapted our approach so that study navigators work only on CRC screening without other unrelated tasks.

A positivity rate of 8–9% is typically reported for patients undergoing a first-time FIT [43, 44]. An aggregate positivity rate of 18% is approximately twofold in excess of this range. We are interested to see if these rates are sustained as the numbers of those screened increase. If high FIT positivity rates are sustained, the colonoscopy outcomes and, if indicated, additional diagnostic studies will be crucial to understanding the causes of excess FIT positivity in AIs.

The CRC screening rates reported for AI/AN vary widely. As noted, the IHS cites national rates of ~ 32% [16] compared to much lower local GPRA rates at facilities participating in the current study. Data from the Behavioral Risk Factor Surveillance System (BRFSS) estimate that an improbable ~ 60% of AI/AN are up-to-date with CRC screening [45, 46]. BRFSS is an annual, state-based, random-digit-dialed telephone survey that includes a series of questions about CRC screening status [47]. The lack of landlines in most households in many AI/AN communities and the lack of cell phone access in many parts of those communities seriously undermine the credibility of BRFSS telephone survey estimates of AI/AN CRC screening rates [48]. Through our REDCap database and improved EHR tracking, we will be well placed to provide accurate CRC screening rates for participating facilities and thereby evaluate the accuracy of GPRA and BRFSS rates.

A limitation of the study is the preliminary nature of the reported screening results; as noted, study navigators have so far reached approximately only 8% of screening-eligible patients at their facilities. Inability at this stage of the project to track CRC screening activity at the individual patient or aggregate levels through the EHR is a further weakness. That said, completion of screening in 37% of patients with whom navigators have interacted compared to baseline GPRA rates of 9% and 13% (and this while the Covid pandemic raged) justifies considerable confidence that navigators using TIMS^©^ will yield major gains in CRC screening rates for AIs. Although to this point, navigators have not encountered any new barriers, we must note that TIMS^©^ messages were elicited from small focus groups and interviews and may not cover the full gamut of beliefs held by all AI tribes.

Another important note is that we gained a thorough understanding of the feasibility of using a type 3 effectiveness-implementation design [35]. A type 3 hybrid design is appropriate only for an intervention that has been thoroughly evaluated for efficacy and is ready to be tested for effectiveness. As noted, we previously demonstrated efficacy of the TIMS^©^ tailored navigation intervention in both a randomized control trial and by non-randomized implementation design conducted in underserved populations [31]. As such, further efficacy testing would be redundant, which creates the opportunity for a novel type 3 design where the primary test is of the implementation processes while also assessing the clinical outcomes anticipated.

## Conclusions

We have demonstrated that a tailored navigator intervention, TIMS^©^, for facilitating CRC screening was readily adapted with provider and community input for application to AIs. A REDCap database for tracking of CRC screening by navigators using tablets or laptops on- or offline is easy to use and allows for generation of aggregate anonymized screening data. Early experience of the intervention tracked with the REDCap database shows promise for increasing CRC screening rates as proposed. Positive FIT rates ≥ twofold in excess of published rates require confirmation but support the urgency of reducing AI CRC screening disparities.

## Supplementary Information

**Additional file 1. **

## Data Availability

Data sharing is not applicable to this article as no datasets were generated or analyzed. The intervention materials (adapted TIMS^©^ message library) and navigator training materials (PowerPoint slides) are available from the corresponding author on request.

## Abbreviations

AI: American Indian
AI/AN: American Indian/Alaska Native
CAB: Community advisory board
CRC: Colorectal cancer
EHR: Electronic health record
FIT: Fecal immunochemical test
IHS: Indian Health Service
IRB: Institutional review board
NCI: National Cancer Institute
TIDieR: Template for Intervention Description and Replication
TIMS^©^: Tailored Intervention Messaging System^©^
US: United States
